# Genome-Based Analysis of Chromosomal Colistin Non-Susceptibility in *Stenotrophomonas pavanii* Isolated from the Phycosphere of *Pectinodesmus pectinatus*

**DOI:** 10.3390/antibiotics15050451

**Published:** 2026-04-30

**Authors:** Heejin Ahn, Hyunwoo Zin, Muhammad Akmal, Tae-Jin Choi

**Affiliations:** 1 Department of Microbiology, Pukyong National University, Busan 48513, Republic of Korea; 2 Forensic DNA Division, National Forensic Service, Wonju 26460, Republic of Korea; 3 Department of Fisheries and Aquaculture, University of Veterinary and Animal Sciences, Lahore 54000, Pakistan

**Keywords:** antimicrobial resistance, colistin non-susceptibility, *Stenotrophomonas pavanii*, phycosphere, whole-genome sequencing, freshwater microalgae

## Abstract

Background/Objectives: Freshwater microalgae–bacteria consortia are increasingly utilized in wastewater treatment and biomass production. However, bacteria associated with the algal phycosphere may act as environmental reservoirs of multidrug-resistant (MDR) phenotypes and antibiotic resistance genes (ARGs), including resistance to last-resort antibiotics such as colistin. Methods: An axenic culture of the freshwater microalga *Pectinodesmus pectinatus* was established using a NaClO-based cleaning protocol. Three phycosphere-associated bacterial strains (*Chryseobacterium* sp., *Pseudomonas monteilii*, and *Stenotrophomonas pavanii*) were isolated and identified by 16S rRNA gene analysis. Antimicrobial susceptibility testing was performed using broth microdilution against 16 antibiotics. Whole-genome sequencing of the most resistant isolate, *S. pavanii*, was conducted using Oxford Nanopore technology, followed by genome annotation and in silico resistome analysis using CARD, AMRFinderPlus, and ResFinder. Results: Among the three isolates, *S. pavanii* exhibited the broadest resistance profile, including high minimum inhibitory concentrations (MICs) to multiple β-lactams and colistin (MIC ≥ 16 μg/mL). No plasmid-borne *mcr* genes were detected. Instead, the genome encoded multiple chromosomal determinants potentially associated with polymyxin non-susceptibility, including lipid A and lipopolysaccharide modification pathways (e.g., *arn* genes and *eptA*), outer-membrane maintenance and LPS transport systems, multidrug efflux pumps, and regulatory elements. Integration of genomic and phenotypic data suggested that the observed colistin non-susceptibility may be associated with intrinsic chromosomal determinants inferred from whole-genome analysis. Conclusions: This study demonstrates that the *P. pectinatus* phycosphere can harbor multidrug-resistant (MDR) bacteria, including strains exhibiting colistin non-susceptibility potentially associated with a repertoire of intrinsic chromosomal resistance mechanisms inferred from genomic analysis. Therefore, freshwater microalgae-based systems should be considered potential environmental reservoirs contributing to the dissemination of antimicrobial resistance.

## 1. Introduction

Microalgae are unicellular, photosynthetic eukaryotic organisms that inhabit terrestrial, freshwater, and marine environments. According to AlgaeBase, approximately 200,000 species of microalgae have been estimated worldwide, and newly described taxa continue to increase [1]. Through chloroplast-based photosynthesis, microalgae utilize carbon dioxide and sunlight to produce organic compounds [2]. Owing to these characteristics, freshwater microalgae-based technologies are increasingly explored as promising bioresources for wastewater treatment, carbon sequestration, biomass production, and the generation of high-value compounds within a circular bioeconomy framework [3].

In large-scale cultivation systems, particularly open or semi-open configurations, microalgal cultures are frequently exposed to contamination by bacteria, fungi, and other microorganisms, often resulting in reduced productivity or culture collapse [4]. However, microalgae do not merely coexist passively with bacteria; instead, they form structured and metabolically active microenvironments known as the phycosphere, in which algal exudates support dense bacterial populations [5]. Interactions within the phycosphere can be beneficial, neutral, or detrimental to algal growth and physiology, but the presence of complex and dynamic bacterial communities complicates the interpretation of algal responses and obscures the roles of individual bacterial taxa [5,6]. Consequently, the establishment of axenic microalgal cultures is considered a critical prerequisite for defining baseline algal characteristics and for systematically investigating specific algal–bacterial interactions [6].

Beyond their influence on algal performance, phycosphere-associated bacterial communities are increasingly recognized as potential environmental reservoirs of antibiotic-resistant bacteria (ARB) and antibiotic resistance genes (ARGs) [7]. Aquatic environments, particularly those influenced by wastewater effluents, agricultural runoff, livestock production, or aquaculture, are subject to chronic antibiotic inputs that impose selective pressure favoring resistant populations [8]. In such settings, biofilms, extracellular polymeric substances, and high cell densities facilitate horizontal gene transfer and the persistence of resistance determinants [9]. Microalgal cultivation systems are enriched in organic carbon and nutrients and promote close physical contact between algae and bacteria, forming microalgae–bacteria consortia that contribute to nutrient cycling and pollutant removal [10]. At the same time, these systems may act as hotspots for the maintenance and dissemination of ARB and ARGs under antibiotic stress [11].

Despite growing recognition of environmental antimicrobial resistance (AMR) risks, integrated studies that combine phenotypic antimicrobial susceptibility testing with genome-based resistance profiling of freshwater phycosphere-associated bacteria remain limited. This gap is particularly evident for non-fermenting Gram-negative bacteria, which often possess extensive intrinsic resistance mechanisms that are difficult to infer from phenotype alone. Within this group, the genus *Stenotrophomonas* is of particular interest. Members of this genus are widely distributed in soil, freshwater, plants, and built environments and are characterized by relatively large genomes, high GC content, and broad intrinsic resistance to multiple antibiotic classes [12,13]. *Stenotrophomonas maltophilia* is well established as an opportunistic multidrug-resistant pathogen in clinical settings [14,15], whereas other species have been predominantly studied in environmental or plant-associated contexts.

*Stenotrophomonas pavanii* was originally described as a nitrogen-fixing endophyte of sugar cane [16] and has subsequently been isolated from freshwater- and plant-associated environments, where it has attracted attention for its metabolic versatility and potential applications in wastewater treatment and polymer degradation [17,18]. However, systematic studies linking whole-genome–based resistance repertoires to phenotypic antimicrobial susceptibility profiles in environmental *S. pavanii* isolates remain scarce. In particular, resistance to colistin, a last-resort polymyxin antibiotic, is poorly characterized in environmental *Stenotrophomonas* species. While plasmid-mediated *mcr* genes are well-recognized drivers of transferable colistin non-susceptibility [19,20], increasing evidence indicates that chromosomally encoded mechanisms—such as lipid A modification, outer-membrane remodeling, regulatory network alterations, and multidrug efflux activity—can mediate high-level colistin non-susceptibility in the absence of *mcr* [21,22,23].

In this study, we established axenic cultures of freshwater microalgae and selected *Pectinodesmus pectinatus* as a robust host species that could be reliably maintained under antibiotic-free conditions. Three phycosphere-associated bacterial strains were isolated from *P. pectinatus*, among which *Stenotrophomonas pavanii* exhibited the broadest multidrug resistance profile and a high colistin minimum inhibitory concentration. We therefore performed whole-genome sequencing of this isolate and integrated in silico resistome prediction with phenotypic MIC data for 16 antibiotics. By systematically analyzing chromosomally encoded resistance determinants, including those associated with lipid A and lipopolysaccharide modification, outer-membrane maintenance, efflux systems, and regulatory pathways, this study aimed to elucidate the genomic basis of multidrug and colistin non-susceptibility in a phycosphere-associated *S. pavanii* strain. Through this integrated approach, we highlight freshwater microalgae-based systems as environmentally relevant reservoirs in the context of antimicrobial resistance dissemination.

## 2. Results

### 2.1. Establishment of Axenic Cultures of Freshwater Microalgae

All ten freshwater microalgal strains used in this study belonged to the phylum *Chlorophyta*, with nine strains assigned to the class *Chlorophyceae* (orders *Sphaeropleales* and *Chlamydomonadales*) and one strain (*Chlorella vulgaris*) to the class *Trebouxiophyceae* (order *Chlorellales*). Following NaClO-based surface-cleaning treatment, fully axenic cultures were successfully established only for *Desmodesmus intermedius* and *Pectinodesmus pectinatus*, both members of the family *Scenedesmaceae*. The remaining eight strains failed to maintain stable growth after cleaning or showed persistent bacterial contamination. Based on growth stability, morphological uniformity, and reproducibility across subcultures, *P. pectinatus* was selected as the representative host for subsequent phycosphere analyses.

### 2.2. Localization of Microbiota and Confirmation of Axenic Status of Pectinodesmus pectinatus

**Figure 1:**
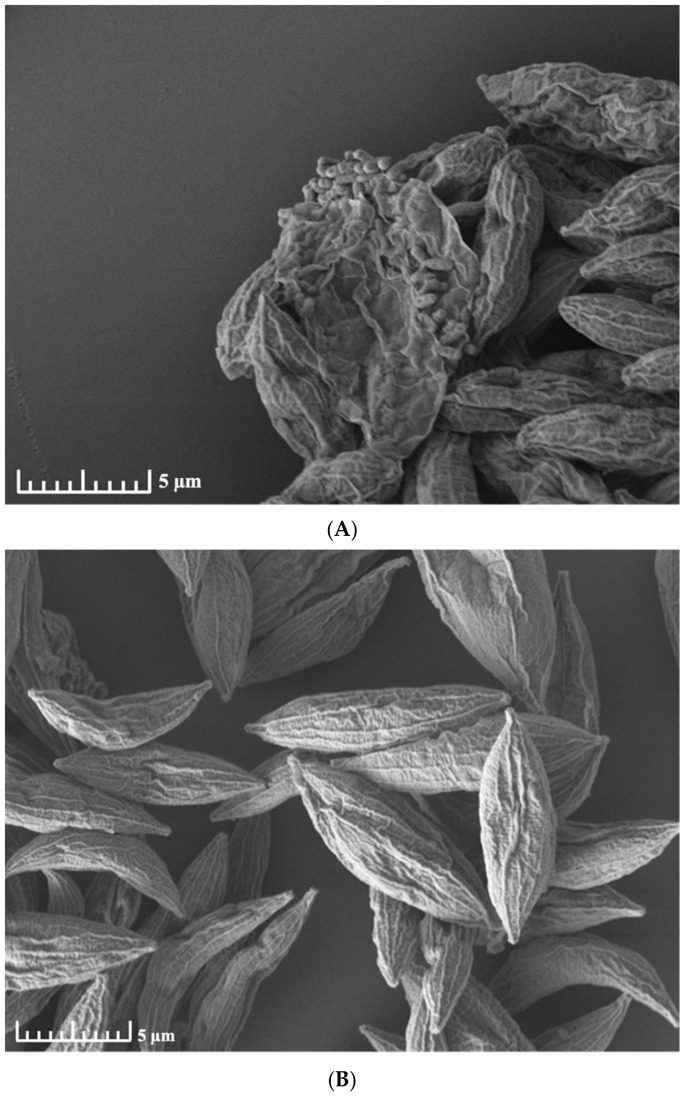
Scanning electron micrographs of *Pectinodesmus pectinatus* before and after NaClO-based cleaning. (**A**) Untreated cells showing aggregated morphology with rough and wrinkled surfaces densely covered by attached bacteria and extracellular matrix-like material. (**B**) NaClO-treated cells appearing as individually separated fusiform units with clearly visible surface ridges and minimal bacterial attachment. Scale bars, 5 μm.

SEM analysis revealed that the bacterial community formed dense, biofilm-like aggregates on the surface of *P. pectinatus* (Figure 1A). Following the NaClO treatment, these bacterial cells were successfully removed, leaving the algal surface smooth and bacteria-free (Figure 1B). Consistent with these observations, no bacterial growth was detected on LB agar plates inoculated with treated cultures, and 16S rRNA gene PCR assays yielded no detectable amplicons, confirming the axenic status of the cultures. This comparative analysis confirmed that the bacterial isolates recovered in this study, including *S. pavanii*, predominantly inhabit the epiphytic phycosphere surface rather than the intracellular compartment. This surface localization is critical for their role in environmental exchange.

### 2.3. Isolation and Taxonomic Identification of Phycosphere-Associated Bacteria

**Figure 2:**
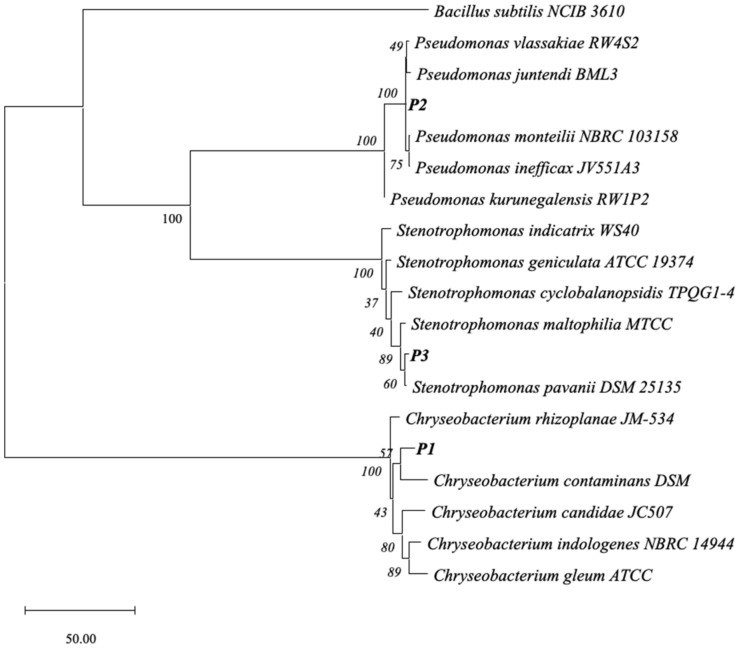
Phylogenetic placement of phycosphere-associated bacterial isolates based on 16S rRNA gene sequences. Neighbor-joining tree showing the relationships of *Chryseobacterium* sp. P1, *Pseudomonas monteilii* P2, and *Stenotrophomonas pavanii* P3 to closely related reference strains. The tree was constructed using MEGA version 12 with 1000 bootstrap replicates; bootstrap values greater than 40% are shown at branch nodes. *Bacillus subtilis* NCIB 3610 was used as an outgroup. Isolates obtained in this study are indicated in bold.

Three distinct bacterial colony morphotypes were recovered from xenic *P. pectinatus* cultures when plated on LB agar. Based on 16S rRNA gene sequence analysis, isolate P1 showed 98.75% sequence identity to *Chryseobacterium candidae* JC507ᵀ, isolate P2 exhibited 99.86% identity to *Pseudomonas monteilii* DSM 14164ᵀ, and isolate P3 showed 99.80% identity to *Stenotrophomonas pavanii* DSM 25135ᵀ. Neighbor-joining phylogenetic analysis confirmed the placement of the three isolates within their respective genera (Figure 2). Genome-based taxonomic analysis further supported the classification of isolate P3 as *S. pavanii*, with an average nucleotide identity of 98.79% and a digital DNA–DNA hybridization value of 90.0% relative to the type strain.

### 2.4. Antimicrobial Susceptibility Profiles of Phycosphere Isolates

Minimum inhibitory concentrations (MICs) for 16 antimicrobial agents were determined for the three phycosphere-associated isolates (Table 1). Because validated species-specific CLSI or EUCAST interpretive criteria are not available, MICs are presented as quantitative values without categorical susceptibility assignments. All three isolates exhibited high MICs to several β-lactam antibiotics, including amoxicillin/clavulanic acid, ampicillin, cefoxitin, and ceftiofur, whereas ceftazidime MICs were comparatively lower. Notably, colistin MICs differed markedly among strains: *Chryseobacterium* sp. P1 and *S. pavanii* P3 exhibited high MICs (≥16 μg/mL; the highest concentration tested), whereas *P. monteilii* P2 remained ≤2 μg/mL. Among non-β-lactam agents, *S. pavanii* displayed elevated MICs to gentamicin, streptomycin, and tetracycline, indicating reduced susceptibility across multiple antibiotic classes.

### 2.5. Genome Assembly and General Features of Stenotrophomonas pavanii P3

Whole-genome sequencing of *Stenotrophomonas pavanii* P3 yielded a closed, single-contig chromosome of 4,540,069 bp with a GC content of 67.17% (Table 2). Genome annotation predicted 4068 coding sequences (CDSs), including 4033 protein-coding genes, as well as 16 rRNA genes and 77 tRNA genes. No plasmids were detected. The assembly consisted of a single contig with an N50 equal to the genome size (4,540,069 bp) and an estimated sequencing depth of approximately 90×. Genome annotation was performed using the NCBI Prokaryotic Genome Annotation Pipeline (PGAP, version 6.10).

### 2.6. Chromosomal Distribution of Putative Antibiotic Resistance Genes in S. pavanii P3

**Figure 3:**
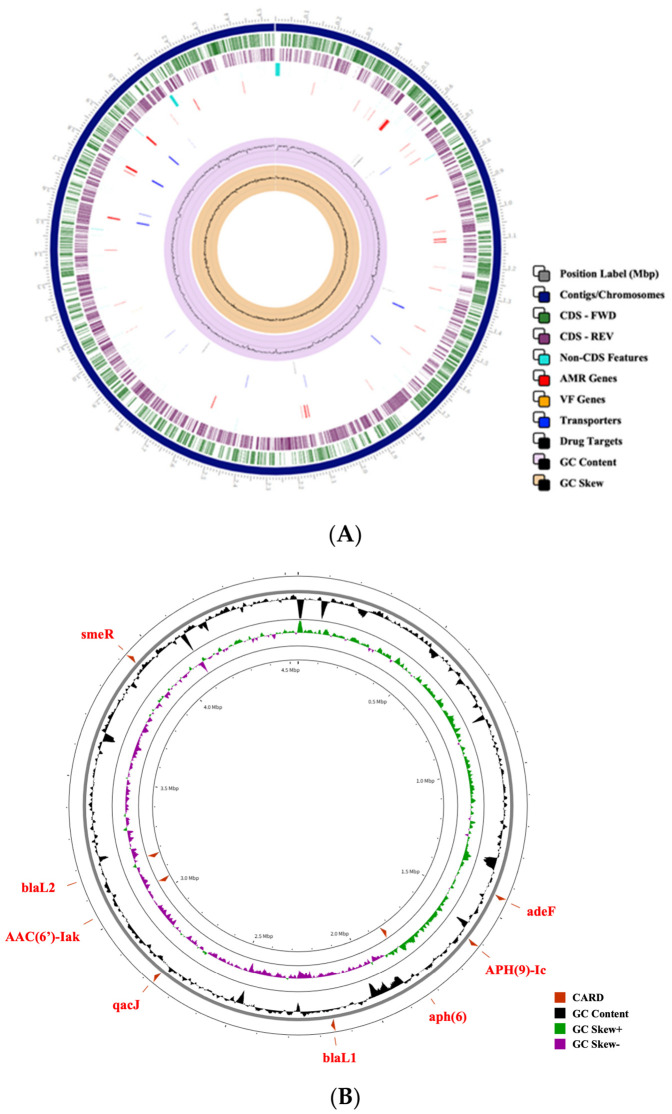
Whole-genome architecture and chromosomal distribution of antibiotic resistance genes in *Stenotrophomonas pavanii* P3. (**A**) Circular genome map showing coding sequences (CDSs) on the forward and reverse strands, predicted antimicrobial resistance (AMR) genes, virulence factor genes, transporters and putative drug targets, followed by GC content and GC skew. (**B**) Genomic locations of chromosomally encoded AMR genes predicted by CARD (version 4.0.1), including β-lactamases (*blaL1*, *blaL2*), aminoglycoside-modifying enzymes, disinfectant resistance gene (*qacJ*), multidrug efflux component (*adeF*), and the efflux regulator *smeR*. No plasmid-mediated *mcr* genes were detected.

Circular genome mapping revealed that the genomic potential for antibiotic resistance genes was distributed throughout the chromosome rather than clustered within a single genomic region (Figure 3A). The genome encoded intrinsic β-lactamase genes (blaL1 and blaL2), aminoglycoside-modifying enzymes, disinfectant resistance genes (qacJ), and components of multidrug efflux systems, including adeF and the efflux regulator smeR (Figure 3B). No plasmid-mediated colistin non-susceptibility (*mcr*) genes (*mcr-1* to *mcr-10*) were identified. Identified resistance genes and their genomic coordinates are summarized in Table 3.

### 2.7. Correlation Between Predicted Genomic Resistome and Phenotypic MIC Profiles

Integration of whole-genome–based resistance predictions with MIC data demonstrated an alignment between genomic potential and observed phenotype profiles (Table 4). The presence of chromosomal β-lactamases and efflux-related determinants corresponded with elevated MICs to penicillins, cephalosporins, and carbapenems. *S. pavanii* P3 exhibited phenotypic resistance to colistin despite the absence of *mcr* genes, prompting further investigation of chromosomally encoded resistance mechanisms. Agents such as trimethoprim–sulfamethoxazole and chloramphenicol showed lower MICs, consistent with the absence of corresponding resistance determinants.

### 2.8. Identification of Chromosomal Determinants Associated with Colistin Non-Susceptibility

Targeted in silico screening provided a descriptive genomic inventory of candidate chromosomal determinants that may support colistin non-susceptibility in *S. pavanii* P3 (Table 5). This repertoire included putative pathways for lipid A modification (*arnBCADTEF*, *eptA*), as well as systems for lipopolysaccharide biosynthesis and transport (*lpx*, *lpt*, *msbA*), outer-membrane maintenance (*mla*), and various regulatory components (*phoP*, *basR*). Additionally, the genome harbors numerous multidrug efflux systems (*acrAB*, *emrAB*, *macAB*, *oqxB7*, *sdrM*, *tolC*), many of which are ubiquitous among Gram-negative bacteria. Several of these determinants were identified in multiple copies, representing a robust chromosomal framework that reflects the genomic potential for intrinsic colistin non-susceptibility in this isolate.

## 3. Discussion

In this study, we established axenic cultures of freshwater microalgae and isolated three phycosphere-associated bacterial strains (*Chryseobacterium* sp., *Pseudomonas monteilii*, and *Stenotrophomonas pavanii*) from *Pectinodesmus pectinatus*. Among these isolates, *S. pavanii* exhibited the broadest multidrug resistance profile and a high colistin minimum inhibitory concentration (MIC). By integrating phenotypic antimicrobial susceptibility testing with whole-genome sequencing (WGS)–based resistome analysis, we systematically characterized the genomic basis of multidrug and colistin non-susceptibility in a phycosphere-associated *S. pavanii* strain and evaluated the concordance between genotype and phenotype.

Application of a NaClO-based surface-cleaning protocol enabled the successful establishment of stable axenic cultures only for *Desmodesmus intermedius* and *P. pectinatus*, both members of the family *Scenedesmaceae*. These species are known to possess thick, multilayered cell walls enriched in algaenan or sporopollenin-like polymers, which likely confer enhanced resistance to oxidative stress during chemical cleaning [24,25]. In contrast, microalgal species lacking such protective cell wall components may be more susceptible to damage under identical treatment conditions, hindering the establishment of axenic cultures. Although direct analysis of cell wall composition and phycosphere community structure was beyond the scope of this study, our observations are consistent with previous reports emphasizing species-specific differences in axenization success [6,24].

The SEM imaging (Figure 1) provided crucial structural context for the isolated *S. pavanii*. The successful removal of bacteria via surface sterilization confirms that this MDR strain is an epiphytic colonizer. Unlike endophytic bacteria that are shielded within the host cell, epiphytic bacteria like *S. pavanii* P3 are directly exposed to the surrounding aqueous environment. This localization likely subjects them to higher selection pressures from dissolved pollutants and antibiotics, potentially driving the accumulation of the robust outer-membrane defense mechanisms (e.g., lipid A modification, efflux pumps) identified in our genomic analysis.

Whole-genome analysis confirmed that *S. pavanii* P3 possesses genomic features typical of the genus *Stenotrophomonas*, including a relatively large genome size, high GC content, and a diverse repertoire of intrinsic resistance determinants [22,23]. Genome-based taxonomic analyses using ANI and digital DNA–DNA hybridization robustly placed the isolate within the species *S. pavanii*, providing a stable taxonomic framework for interpreting its resistance traits. While *S. pavanii* has primarily been investigated for its ecological functions and biotechnological potential in environmental systems [16,17,18], comparatively little is known about its antibiotic resistance characteristics. Our findings extend previous observations by demonstrating that environmental *S. pavanii* isolates can harbor resistance phenotypes and genomic architectures comparable to those documented in other Stenotrophomonas species, highlighting their potential ecological significance in resistance dissemination.

Phenotypic antimicrobial susceptibility testing revealed elevated MICs to multiple β-lactam antibiotics, aminoglycosides, tetracycline, and colistin in *S. pavanii* P3 (Table 1). These profiles were largely concordant with the presence of chromosomally encoded resistance determinants identified by WGS, including the intrinsic β-lactamases *blaL1* and *blaL2*, aminoglycoside-modifying enzymes, and multiple efflux-related genes (Table 3). Consistent with previous reports on *Stenotrophomonas* spp., these intrinsic mechanisms are consistent with the broad multidrug-resistant phenotype observed in this isolate [26,27,28]. Notably, although the ceftazidime MIC was comparatively low, categorical susceptibility interpretation was not assigned because the CLSI has removed ceftazidime breakpoints for *Stenotrophomonas* spp. due to insufficient correlation between in vitro MICs and clinical outcomes [29]. Variation in β-lactam MICs among cephalosporins may reflect differential regulation or allelic diversity of β-lactamases, as well as the influence of efflux systems and testing conditions [30,31].

A key finding of this study is the high colistin MIC observed in *S. pavanii* P3 despite the absence of plasmid-mediated *mcr* genes (Table 4). While *mcr* genes are recognized as major drivers of transferable colistin non-susceptibility [19,20], increasing evidence indicates that chromosomally encoded mechanisms can mediate stable and high-level colistin non-susceptibility in Gram-negative bacteria [21,22,23]. In this context, WGS analysis revealed that *S. pavanii* P3 harbors a complete set of genes associated with lipid A modification, including the *arnBCADTEF* operon and multiple copies of *eptA* (Table 5). However, the presence of multiple copies of *eptA* observed in this study may reflect either true gene duplication or potential assembly artifacts in repetitive regions, which cannot be fully resolved without additional validation. In other Gram-negative species, these pathways are known to involve the addition of 4-amino-4-deoxy-L-arabinose and phosphoethanolamine to lipid A. This modification reduces the net negative charge of the outer membrane, which potentially weakens electrostatic interactions with polymyxins, suggesting a putative mechanism for high MICs observed in strain P3 [21,22,23,32,33,34]. Similar mechanisms have been implicated in intrinsic polymyxin resistance in *S. maltophilia* and other non-fermenting Gram-negative bacteria [22,35].

In addition to lipid A modification, *S. pavanii* P3 encoded a comprehensive set of genes involved in lipopolysaccharide (LPS) biosynthesis, transport, and outer-membrane maintenance, including *lpx*, *lpt*, *msbA*, and *mla* gene clusters (Table 5). These systems are essential for maintaining outer-membrane integrity and permeability barriers targeted by polymyxins [36,37,38]. The presence of these biosynthetic and transport clusters suggests a genomic capacity for envelope remodeling, which has been associated with colistin non-susceptibility in related taxa.

**Figure 4:**
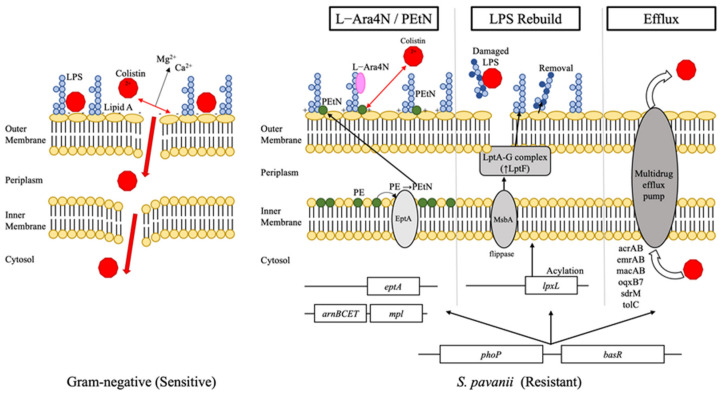
Proposed chromosomal mechanisms underlying colistin non-susceptibility in *Stenotrophomonas pavanii* P3. Schematic representation of chromosomally encoded resistance strategies inferred from whole-genome analysis. In colistin-susceptible Gram-negative bacteria (**left**), colistin binds to negatively charged lipid A, disrupting outer-membrane integrity. In *S. pavanii* P3 (**right**), colistin non-susceptibility is proposed to involve (i) lipid A modification via L-Ara4N and/or phosphoethanolamine addition (*arnBCADTEF*, *eptA*), reducing net negative charge; (ii) lipopolysaccharide biosynthesis, transport, and outer-membrane maintenance systems (*lpx*, *lpt*, *msbA*, *mla*); and (iii) multidrug efflux pumps and regulatory networks that collectively limit colistin binding and penetration. All depicted elements represent predicted genomic determinants rather than experimentally validated mechanisms. Red hexagons indicate colistin molecules and arrows indicate the proposed direction of colistin movement, lipid A modification, LPS transport/removal, and efflux-mediated extrusion.

Furthermore, genomic analysis identified several multidrug efflux systems and regulatory components that may influence antibiotic susceptibility by modulating intracellular drug accumulation and envelope stress responses [35,39,40]. Here, we emphasize systems with documented or plausible relevance to polymyxin tolerance, particularly those linked to envelope stress adaptation and outer membrane homeostasis, which may indirectly affect susceptibility to membrane-targeting agents such as colistin. In contrast, broadly conserved efflux pumps, including *acrAB* and *tolC*—widely distributed across Gram-negative bacteria and primarily associated with general physiological functions—are reported as part of the genomic inventory without implying a direct mechanistic role in high-level colistin non-susceptibility. Unlike lipid A modification pathways, which have established roles in polymyxin resistance, efflux-mediated polymyxin export in *Stenotrophomonas* remains insufficiently characterized. Although efflux alone is unlikely to explain the observed phenotype, its contribution within a multilayered resistance network cannot be excluded and warrants further functional validation. Taken together, these findings support a model of potential colistin non-susceptibility in *S. pavanii*, where a multilayered chromosomal repertoire of lipid A modification, LPS transport, and efflux systems likely provides a robust defense against polymyxin binding and penetration. Similar integrative resistance architectures have been described in *S. maltophilia* and other non-fermenting Gram-negative bacteria, where intrinsic envelope remodeling and regulatory responses play central roles in polymyxin resistance in the absence of plasmid-mediated *mcr* genes [21,22,23,35,39]. A schematic overview of the proposed chromosomal mechanisms inferred from whole-genome analysis is provided in Figure 4 to summarize the relationships among these resistance modules.

The phycosphere context of this isolate has important environmental implications. Microalgae-based cultivation systems are increasingly proposed for wastewater treatment and resource recovery, yet they create nutrient-rich microenvironments that support dense bacterial colonization and close cell-to-cell contact [10,41,42]. Such conditions may facilitate the environmental persistence and spread of antibiotic-resistant bacteria and resistance determinants within microalgae-based systems [11,43]. The identification of a multidrug-resistant, colistin non-susceptible *S. pavanii* strain within the *P. pectinatus* phycosphere illustrates the potential for freshwater microalgae-based systems to harbor resistant bacteria. While this study focuses on a specific isolate from a single host, it underscores the importance of considering antimicrobial resistance risks in the design and management of such biotechnological systems.

Several limitations of this study should be acknowledged. While the whole-genome analysis of *S. pavanii* P3 revealed a comprehensive suite of genes typically associated with colistin non-susceptibility, such as the *arnBCADTEF* operon and eptA homologs, it is important to acknowledge the inherent limitations of in silico attribution. The presence of these genes highlights a significant genomic potential for resistance, but it does not serve as direct evidence of their functional expression or their actual biochemical impact on the cell. In this study, we did not perform transcriptional profiling (e.g., RT-qPCR) or biochemical confirmation of lipid A modifications (e.g., via MALDI-TOF MS). Therefore, these chromosomal elements should be considered putative drivers of the observed phenotype rather than confirmed causes.

Phenotypic susceptibility testing was conducted under standardized laboratory conditions, and MIC determinations were performed twice independently. Although broth microdilution reduces methodological variability, colistin MIC testing is known to be sensitive to experimental conditions and should be interpreted within the applied testing framework. Future research involving gene knockout models, membrane lipidomics, gene expression analysis, and biochemical characterization of lipid A modifications will be essential to definitively bridge the gap between these genomic blueprints and the high-level colistin non-susceptibility observed in phycosphere-associated Stenotrophomonas.

Although this study focused on a single *S. pavanii* isolate, previous genomic studies of *Stenotrophomonas* species, particularly *S. maltophilia*, have reported similar intrinsic resistance mechanisms, including lipid A modification pathways and multidrug efflux systems [22,44,45]. This suggests that the resistance features observed in *S. pavanii* P3 may not be entirely strain-specific, but rather reflect conserved genomic traits within the genus. However, further comparative genomic analyses across multiple *S. pavanii* strains would be required to confirm the generality of these findings.

The *Chryseobacterium* sp. P1 isolate identified in this study showed relatively low 16S rRNA gene sequence identity (98.75%) to its closest type strain (Figure 2), suggesting that it may represent a potentially novel taxon. In addition, this isolate exhibited a high colistin MIC (≥16 μg/mL), indicating potential relevance to antimicrobial resistance. However, as the primary focus of this study was the genomic characterization of *S. pavanii* P3, further genomic and phenotypic characterization of P1 was beyond the scope of the present work. Future studies, including whole-genome sequencing and taxonomic characterization, will be necessary to clarify the biological and taxonomic significance of this isolate.

In summary, this study demonstrates that a phycosphere-associated *Stenotrophomonas pavanii* isolate from freshwater microalgae exhibits multidrug resistance and high-level colistin non-susceptibility. This phenotype is associated with chromosomally encoded mechanisms in the absence of *mcr* genes. By integrating WGS-based resistome analysis with phenotypic susceptibility data, our findings provide insight into non-*mcr* strategies of polymyxin non-susceptibility and highlight freshwater microalgae-based systems as environmentally relevant reservoirs in the broader context of antimicrobial resistance.

## 4. Materials and Methods

### 4.1. Microalgal Strains and Culture Conditions

Ten freshwater microalgal strains were obtained from the Nakdonggang National Institute of Biological Resources (NNIBR, Sangju, Republic of Korea). All strains were supplied as xenic (non-axenic) cultures and were activated according to the supplier’s standard protocols. The strains were cultivated in Bold’s Basal Medium (BBM), which was sterilized by autoclaving at 121 °C for 15 min prior to use. Cultures were maintained at 20 °C under continuous illumination (50 μmol photons m^−2^ s^−1^) or in the dark, as specified, and were subcultured every two weeks to sustain active growth.

### 4.2. Establishment of Axenic Pectinodesmus pectinatus Cultures and Microscopy

Two-week-old cultures of *Pectinodesmus pectinatus* were harvested by centrifugation at 3500 rpm for 10 min. Cell pellets were resuspended in 900 μL of distilled water, followed by the addition of 100 μL of 1% (*v*/*v*) sodium hypochlorite (NaClO) to achieve a final concentration of 0.1% (*v*/*v*). The suspension was mixed by inversion and incubated for 105 s. Cells were then centrifuged at 2000× *g* for 2 min, washed twice with distilled water, and resuspended in fresh BBM. Treated cultures were incubated under standard growth conditions.

Axenic status was assessed by spreading aliquots of treated cultures onto LB agar plates to test for bacterial growth and by PCR amplification of the 16S rRNA gene. For localization of the phycosphere-associated microbiota and confirmation of axenic status, untreated and NaClO-treated algal cells were fixed with 2.5% paraformaldehyde and 2.5% glutaraldehyde in 0.05 M sodium cacodylate buffer, post-fixed with 1% osmium tetroxide, dehydrated through a graded ethanol series, treated with hexamethyldisilazane, sputter-coated with gold, and observed using a field-emission scanning electron microscope (FE-SEM; MIRA3 LMH, TESCAN, Brno, Czech Republic).

### 4.3. Isolation and Identification of Phycosphere-Associated Bacteria

Phycosphere-associated bacteria were isolated by spreading 100 μL of xenic *P. pectinatus* cultures onto LB agar plates, followed by incubation at 37 °C for 24 h. This temperature was specifically chosen to select for bacteria capable of surviving at human physiological temperatures, thereby highlighting those with potential clinical or zoonotic relevance. Colonies with distinct morphologies were selected and purified by repeated streaking. Genomic DNA was obtained directly from colonies and used as template for PCR amplification of the 16S rRNA gene using universal primers 27F and 1492R. PCR amplification was performed using EzPCR™ FAST 5× Master Mix (ELPIS-BIOTECH, Daejeon, Republic of Korea) under rapid-cycling conditions recommended by the manufacturer. Amplicons were verified by agarose gel electrophoresis and visualized under UV illumination.

### 4.4. Antimicrobial Susceptibility Testing

Minimum inhibitory concentrations (MICs) of three bacterial isolates (*Chryseobacterium* sp., *Pseudomonas monteilii*, and *Stenotrophomonas pavanii*) were determined using the Sensititre broth microdilution system with the KRNV5F panel (Thermo Fisher Scientific, Waltham, MA, USA). Isolates were cultured on tryptone soya agar at 37 °C (to assess potential clinical and zoonotic relevance) for 24 h, suspended in 0.85% saline, and adjusted to a 0.5 McFarland standard. Standardized suspensions were diluted in cation-adjusted Mueller–Hinton broth containing TES buffer and dispensed into microdilution plates using the Sensititre AIM system. Plates were incubated at 37 °C for 18–24 h, to maintain consistency with standard CLSI/NARMS clinical protocols [46,47], and MIC values were read using the Sensititre OptiRead system. This approach enables the direct benchmarking of environmental isolates against established antimicrobial resistance data for human pathogens. Antimicrobial agents and concentration ranges were selected according to CLSI guidelines and the NARMS Human Isolates Report [46,47]. Because species-specific interpretive criteria were unavailable, MICs are presented as quantitative values. Colistin susceptibility was assessed using the KRNV5F panel, which applies a standardized broth microdilution format and includes colistin within the recommended testing range. MIC testing for each isolate was performed independently on two separate occasions, and consistent results were obtained. Each microdilution plate contained internal positive growth control wells to confirm bacterial viability and assay performance.

### 4.5. Phylogenetic Analysis Based on 16S rRNA Gene Sequences

16S rRNA gene sequences were aligned using MEGA version 12. Phylogenetic trees were constructed using the neighbor-joining method, and branch support was evaluated by bootstrap analysis with 1000 replicates. *Bacillus subtilis* NCIB 3610 was used as an outgroup.

### 4.6. Whole-Genome Sequencing and Assembly of Stenotrophomonas pavanii

Genomic DNA of *S. pavanii* P3 was extracted using the DNeasy Blood & Tissue Kit (Qiagen, Hilden, Germany). DNA quantity and quality were assessed using a NanoDrop spectrophotometer (Thermo Fisher Scientific, Waltham, MA, USA) and a Qubit 4 fluorometer (Invitrogen, Carlsbad, CA, USA). Whole-genome sequencing was performed on the Oxford Nanopore Technologies platform using a MinION Mk1C device and a Flongle R9.4.1 flow cell from Oxford Nanopore Technologies (Oxford, UK). Reads were de novo assembled using Flye v2.9.1 and polished with Medaka v1.7.2. Assembly quality metrics were assessed using QUAST v5.3.0 [48]. Subsequently, genome annotation was conducted using Prokka v1.14.6 [49].

### 4.7. Genome-Based Taxonomic and Resistome Analyses

Genome-based taxonomic assignment was performed using the Type Strain Genome Server (TYGS). Average nucleotide identity (ANI) was calculated using OrthoANI [50] implemented in EzBioCloud. Antibiotic resistance genes were predicted using AMRFinderPlus (version 3.12.8), the Resistance Gene Identifier (RGI, version 6.0.5) of the Comprehensive Antibiotic Resistance Database (CARD, version 4.0.1), and ResFinder (version 4.7.2). For CARD analysis, only strict hits were considered. Sequence similarity thresholds of ≥90% identity and ≥60% coverage were applied for AMRFinderPlus and ResFinder analyses. Results from the three tools were integrated to generate a comprehensive resistome profile. Screening for plasmid-mediated colistin resistance (*mcr*) genes was performed using these tools, which collectively include all currently described *mcr* variants (*mcr*-1 to *mcr*-10), with default database parameters and sequence alignment thresholds applied for detection. Chromosomal determinants associated with colistin non-susceptibility were identified by targeted screening for genes involved in lipid A modification, lipopolysaccharide biosynthesis and transport, efflux systems, and regulatory pathways, as described previously [21,22,23].

## 5. Conclusions

In this study, we established an axenic culture of the freshwater microalga *Pectinodesmus pectinatus* to characterize its phycosphere-associated bacteria, with a specific focus on a multidrug-resistant *Stenotrophomonas pavanii* isolate exhibiting high-level colistin non-susceptibility. By integrating phenotypic antimicrobial susceptibility testing with whole-genome sequencing (WGS) resistome analysis, we found that this resistance profile may be associated with a repertoire of chromosomally encoded determinants inferred from whole-genome analysis, rather than plasmid-borne *mcr* genes.

The identified genomic features include pathways for lipid A and lipopolysaccharide modification, outer-membrane maintenance, and multidrug efflux pumps regulatory components consistent with genomic features previously associated with intrinsic resistance frameworks in other Gram-negative bacteria. While the elevated colistin Minimum Inhibitory Concentration (MIC) was experimentally confirmed, we noted that these specific resistance mechanisms are inferred from genomic data and require future functional validation.

Our findings illustrate that phycosphere-associated bacteria can harbor resistance phenotypes and genomic architectures comparable to those found in clinically relevant opportunistic pathogens. The presence of such a strain within a freshwater microalgal environment underscores the need to evaluate microalgae-based cultivation systems as potential environmental reservoirs for antimicrobial resistance (AMR).

Ultimately, this work demonstrates the value of combining axenic culturing with genome-based analyses to investigate resistance dynamics in environmental bacteria. As microalgae-based biotechnological frameworks expand, it is essential that AMR risk assessments and continued surveillance are incorporated into their design and management to clarify their role in the global dissemination of resistance.

## Figures and Tables

**Table 1:** Minimum inhibitory concentration (MIC) profiles of three phycosphere-associated bacterial isolates against 16 antibiotics.

No.	Antibiotics	Class	*Chryseobacterium* sp.	*P. monteilii*	*S. pavanii*
1	Amoxicillin/Clavulanic Acid	β-lactam—Penicillin	>32	>32	>32
2	Ampicillin	β-lactam—Penicillin	>64	>64	>64
3	Cefepime	β-lactam—4th-gen Cephalosporin	>16	2	16
4	Cefoxitin	β-lactam—2nd-gen Cephalosporin	>32	>32	>32
5	Ceftazidime	β-lactam—3rd-gen Cephalosporin	2	4	4
6	Ceftiofur	β-lactam—3rd-gen Cephalosporin	>8	>8	>8
7	Chloramphenicol	Phenicol	8	32	16
8	Ciprofloxacin	Fluoroquinolone	0.25	≤0.12	2
9	Colistin	Polymyxin	>16	≤2	>16
10	Gentamicin	Aminoglycoside	2	≤1	64
11	Meropenem	Carbapenem	>4	4	>4
12	Nalidixic Acid	Quinolone—1st gen	16	64	8
13	Streptomycin	Aminoglycoside	≤16	≤16	>128
14	Sulfisoxazole	Sulfonamide	256	>256	≤16
15	Tetracycline	Tetracycline	8	≤2	16
16	Trimethoprim/Sulphamethoxazole	Sulfonamide (Combo)	0.25	>4	≤0.12

**Table 2:** Genome features of *Stenotrophomonas pavanii* P3.

Features	Values
Genome size (bp)	4,540,069
No. of contigs	1
N50 (bp)	4,540,069
GC content (%)	67.17%
No. of CDSs	4068
No. of rRNA genes (5 S, 16 S, 23 S)	16 (6, 5, 5)
No. of tRNA gene	77
Pseudo genes	35
GenBank accession number	CM148750.1
Annotation pipeline	NCBI PGAP (v6.10)

**Table 3:** Antibiotic resistance genes identified in *Stenotrophomonas pavanii* P3 based on CARD (version4.0.1), AMRFinderPlus (version 3.12.8), and ResFinder (version 4.7.2) analyses.

Gene	Antibiotic Class	Resistance Mechanism	Start-End	Database (Tool)
*AAC(6′)-Iak*	aminoglycoside antibiotic	antibiotic inactivation	3,040,612–3,041,073	CARD, ResFinder, AMRFinderPlus
*adeF*	fluoroquinolone antibiotic, tetracycline antibiotic	antibiotic efflux	1,444,311–1,447,481	CARD
*aph(6)*	aminoglycoside antibiotic	antibiotic inactivation	1,833,768–1,834,574 (−strand)	AMRFinderPlus
*APH(9)-Ic*	aminoglycoside antibiotic	antibiotic inactivation	1,617,904–1,618,911	CARD
*blaL1*	carbapenem, cephalosporin, penicillin beta-lactam	antibiotic inactivation	2,156,120–2,156,986	AMRFinderPlus
*blaL2*	cephalosporin, penicillin beta-lactam	antibiotic inactivation	3,164,653–3,165,555 (−strand)	AMRFinderPlus
*qacJ*	disinfecting agents and antiseptics	antibiotic efflux	2,771,777–2,772,109	CARD
*smeR*	aminoglycoside antibiotic, cephalosporin, penicillin beta-lactam	antibiotic efflux	3,930,278–3,930,967	CARD

**Table 4:** Comparative overview of genomic resistance determinants and phenotypic MIC profiles in *Stenotrophomonas pavanii* P3.

No.	Antibiotics	MIC	Gene	Putative Genomic Basic
1	Amoxicillin/Clavulanic Acid	>32	+	L1/L2 β-lactamases
2	Ampicillin	>64	+	L1/L2 β-lactamases
3	Cefepime	16	+	L1/L2 + efflux
4	Cefoxitin	>32	+	Cephamycins inactive
5	Ceftazidime	4	+	CLSI breakpoints removed
6	Ceftiofur	>8	+	No activity vs. genus
7	Chloramphenicol	16	−	CLSI breakpoint
8	Ciprofloxacin	2	+	EUCAST PK/PD cutoff exceeded
9	Colistin	>16	−	Outer-membrane features
10	Gentamicin	64	+	Aminoglycoside impermeability
11	Meropenem	>4	+	L1 metallo-β-lactamase
12	Nalidixic Acid	8	+	Poor activity vs. genus
13	Streptomycin	>128	+	Aminoglycoside resistance
14	Sulfisoxazole	≤16	−	Sulfonamide activity preserved
15	Tetracycline	16	+	Above typical PK/PD targets
16	Trimethoprim/ Sulphamethoxazole	≤0.12	−	CLSI breakpoint

The shaded low MIC to Ceftazidime and resistance to colistin are discussed in the main text below.

**Table 5:** Genomic inventory of candidate chromosomal determinants putatively associated with envelope modification, regulatory signaling, and multidrug efflux in *Stenotrophomonas pavanii* P3.

Category	Representative Gene	Copy No.	Category	Representative Gene	Copy No.
L-Ara4N/ PEtN	*arnB*	1	LPS biosynthesis/transport	*lptF*	1
	*arnC*	1		*lptG*	1
	*arnE*	2		*lpxK*	1
	*arnT*	2		*lpxL*	2
	*ctaB*	1		*msbA*	3
	*eptA*	2		*waaA*	1
	*ispA*	1	Regulators	*phoP*	5
	*ispU*	1		*basR*	3
	*mpl*	1	Efflux	*acrA*	1
LPS biosynthesis/transport	*kdsA*	1		*acrB*	2
	*kdsB*	1		*emrA*	4
	*kdsC*	1		*emrB*	2
	*kdsD*	1		*macA*	3
	*lptA*	1		*macB*	3
	*lptB*	1		*oqxB7*	1
	*lptC*	1		*sdrM*	1
	*lptD*	1		*tolC*	1
	*lptE*	1			

## Data Availability

The complete genome sequence of *Stenotrophomonas pavanii* P3 has been deposited in GenBank under accession number CM148750.1 within BioProject PRJNA1347964. The raw Nanopore sequencing reads are available in the NCBI Sequence Read Archive (SRA) under accession number SRR37939269. The 16S rRNA gene sequences of isolates P1, P2, and P3 have been deposited in GenBank under accession numbers PZ247568, PZ247569, and PZ247570, respectively. The *S. pavanii* P3 strain has been deposited in the Freshwater Bioresources Collection (FBCC) of the Nakdonggang National Institute of Biological Resources under accession number FBCC-B22969.
